# Loss of smelling is an early marker of aging and is associated with inflammation and DNA damage in C57BL/6J mice

**DOI:** 10.1111/acel.13793

**Published:** 2023-02-27

**Authors:** Xiuli Dan, Beimeng Yang, Ross A. McDevitt, Samuel Gray, Xixia Chu, Quia Claybourne, David M. Figueroa, Yongqing Zhang, Deborah L. Croteau, Vilhelm A. Bohr

**Affiliations:** ^1^ Section on DNA Repair National Institute on Aging, NIH Maryland Baltimore USA; ^2^ Comparative Medicine Section National Institute on Aging, NIH Baltimore Maryland USA; ^3^ Laboratory of Genetics and Genomics National Institute on Aging, National Institutes of Health Baltimore Maryland USA; ^4^ Danish Center for Healthy Aging University of Copenhagen Copenhagen Denmark

**Keywords:** aging, DNA damage, inflammation, NAD^+^, smelling loss

## Abstract

Olfactory dysfunction is a prevalent symptom and an early marker of age‐related neurodegenerative diseases in humans, including Alzheimer's and Parkinson's Diseases. However, as olfactory dysfunction is also a common symptom of normal aging, it is important to identify associated behavioral and mechanistic changes that underlie olfactory dysfunction in nonpathological aging. In the present study, we systematically investigated age‐related behavioral changes in four specific domains of olfaction and the molecular basis in C57BL/6J mice. Our results showed that selective loss of odor discrimination was the earliest smelling behavioral change with aging, followed by a decline in odor sensitivity and detection while odor habituation remained in old mice. Compared to behavioral changes related with cognitive and motor functions, smelling loss was among the earliest biomarkers of aging. During aging, metabolites related with oxidative stress, osmolytes, and infection became dysregulated in the olfactory bulb, and G protein coupled receptor‐related signaling was significantly down regulated in olfactory bulbs of aged mice. Poly ADP‐ribosylation levels, protein expression of DNA damage markers, and inflammation increased significantly in the olfactory bulb of older mice. Lower NAD^+^ levels were also detected. Supplementation of NAD^+^ through NR in water improved longevity and partially enhanced olfaction in aged mice. Our studies provide mechanistic and biological insights into the olfaction decline during aging and highlight the role of NAD^+^ for preserving smelling function and general health.

## Graphical abstract

In the present study, the smelling loss of C57BL/6J mice during non‐pathological aging was systematically characterized. Our data showed that smelling changes were among the earliest changed behaviors during aging. Metabolites related with oxidative stress, osmolytes, and infection were dysregulated in brain regions related with olfaction. DNA damage markers, inflammation, and glial activation increased significantly in OBs of older mice, which together contributed to olfaction decline. Supplementation of NAD^+^ improved longevity and partially enhanced olfaction in aged mice.

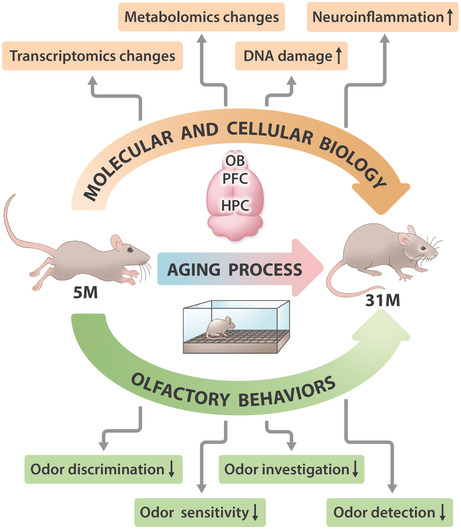

**Abbreviations**
DG: dentategyrus
GCL: granule cell layer
GL: glomerular layer
GO: gene ontology
HPC: hippocampus
NAD^+^
: nicotinamideadenine dinucleotide
NR: nicotinamide riboside
OB: olfactory bulb
PARylation: ADP‐ribosylation
PFC: prefrontal cortex

## 1 INTRODUCTION

The prevalence of olfactory dysfunction increases significantly in older individuals, as more than 50% of people at 65–80 years old experience decreased ability to smell (Palmquist et al., 2020; Seubert et al., 2017). The loss of olfaction has a profoundly negative impact on patients' reported enjoyment of food and quality of life; however, no treatments are available for olfactory dysfunction (Daramola & Becker, 2015).

An increasing body of evidence suggests that olfactory dysfunction is an early biomarker for Alzheimer's and Parkinson's diseases, appearing prior to cognitive and motor dysfunction (Dan et al., 2021; Murphy, 2019). However, olfactory dysfunction can also occur in aged humans and animals in the absence of a disease‐associated pathology. Thus, it is important to distinguish olfactory dysfunction linked to nonpathological aging from disease‐associated pathology. This is a challenge in human populations, where there are limited tools for identifying prodromal neurodegenerative diseases.

Various animal models have been used to study olfaction, including mice, rats, *Caenorhabditis elegans*, and fruit flies. Among these, the olfactory system of mice and rats is structurally and functionally most similar to the olfactory system in humans (Dan et al., 2021). In animal models, well‐characterized assays are available to assess several domains of olfactory function, including odor detection, odor sensitivity, odor discrimination, and odor habituation (Dan et al., 2021). Although aging‐related olfactory dysfunction has been investigated in prior studies using various strains of rodent models (Kraemer & Apfelbach, 2004; Patel & Larson, 2009), a majority of these prior studies only assess one or two domains of olfaction; furthermore, in most of these studies the inclusion of only a single “aged” time point prevented assessment of progressive aspects of olfactory loss throughout aging. Thus, it is not known whether different domains of olfactory function decline at different ages in mice.

Nicotinamide adenine dinucleotide (NAD^+^) is a coenzyme for redox reactions and an essential cofactor for non‐redox NAD^+^‐dependent enzymes which plays vital roles in DNA repair, cell metabolism, and cell survival (Covarrubias et al., 2021). The abundance of NAD^+^ declines gradually in animals and humans with age, which may contribute to age‐associated cognitive decline, cancer risk, and increased susceptibility to age‐related disease (Covarrubias et al., 2021). Dosing with supplemental NAD^+^ has been explored in normal aging and specific age‐related diseases. Despite reports of NAD^+^ decline in aging, NAD^+^ has been intensively investigated in hippocampal brain tissue, skeletal muscle, adipose tissue, and liver, but not studied in many other tissues (Peluso et al., 2021). To date, there is little information on NAD^+^ abundance in the olfactory bulb, changes with age, and its response to supplementation with NAD^+^.

Here, we used wild‐type C57BL/6J mice, a commonly used mouse strain for aging research, to systematically investigate olfaction decline during aging. We tracked the changes of olfactory function in different domains across the lifespan (mature, middle age, old age, and advanced old age), comparing their time courses with other cognitive and motor changes in aging. We explored molecular mechanisms that drive olfactory dysfunction, and we tested the effects of NAD^+^ analog nicotinamide riboside (NR) on olfactory function in aged mice.

## 2 RESULTS

### 2.1 Aged mice show deficits in olfactory functions

C57BL/6J mice at mature age (5M), middle age (13M), old age (21M) and advanced age (31M) were used in the present study. Significant weight gain was observed from 5 to 21 months of age, while animal weight decreased from 21 to 31 months of age (Figure S1A), similar to changes during mid‐ and late life stages of humans. Interestingly, male mice had a higher weight than age‐matched female mice in the 5, 13 and 21M groups, while no significant difference in weight was observed in males and females at 31M (Figure S1B).

**FIGURE 1:**
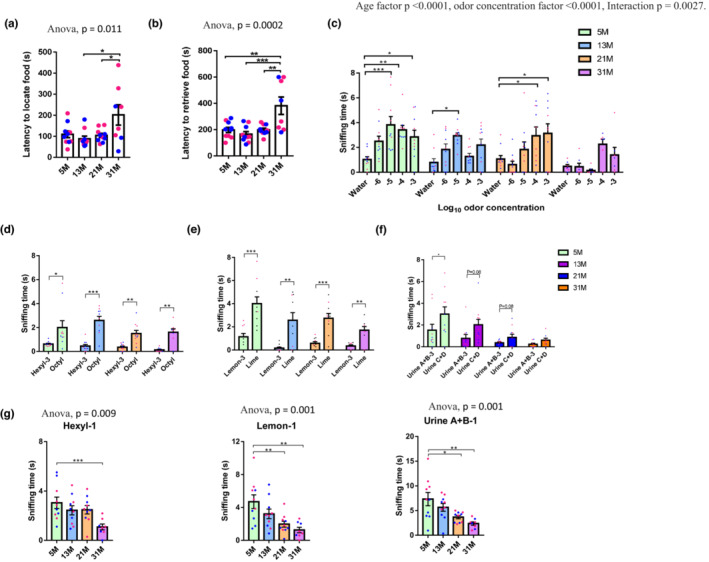
Aged mice show deficits in odor detection, sensitivity, and discrimination. (a, b) Time to locate (a) and retrieve food (b) in the buried food test. One‐way ANOVA followed by Tukey's multiple comparisons test was applied for statistical analysis. *N* = 8–10 mice per group. (c) Odor sensitivity test. Two‐way ANOVA followed by Turkey's multiple comparison test was applied for multiple comparison. The average sniffing time on the last two presentations of water are plotted. *N* = 7–10 mice per group. (d–f) Discrimination of similar odor pairs in the habituation–dishabituation task. Paired *t* tests (two‐tailed) were performed to compare the sniffing time between the third presentation of odor A (A‐3) and subsequent presentation of odor B. Odor pairs include hexyl acetate and octyl acetate (d), lemon and lime extracts (e) and mouse urine pools (f). *N* = 8–10 mice per group. (g) Time investigating novel odorants. One‐way ANOVA was applied for statistical analysis. *N* = 8–10 mice per group. Blue and red dots, respectively, represent male and female mice for all graphs. Values are mean and SEM, *p < 0.05; **p < 0.01; ***p < 0.001.

To evaluate olfactory function, we performed several well‐characterized behavioral assays to assess the four major domains of olfaction which include odor detection, sensitivity, discrimination, and habituation for different odors. In the buried food test, the time required for a food‐deprived mouse to find and grasp a hidden/buried piece of food was recorded to measure odor detection. Our results showed that aged mice spent a significantly longer time before digging in the correct location (Figure 1a) and to retrieve the buried food (Figure 1b). There were no sex differences in the buried food test. There were no significant differences in total distance traveled in the open field across age groups (Figure S1C), suggesting that the differences in the buried food test were not due to mobility but specific to odor detection ability.

To assess odor sensitivity, the responses of mice to dilutions of orange extract were investigated. As shown in Figure 1c, 5M and 13M mice were able to differentiate water from orange extract diluted 10^5^‐fold, while the sensitivity threshold was worse for mice older than 13M. Specifically, the threshold for 21M mice was 10^4^‐fold dilution, while 31M mice could not detect the odorant at the highest tested concentration (10^3^‐fold dilution). These results show that there is an age‐dependent decline of odor sensitivity. In addition, 5M females showed higher odor sensitivity (lower concentration threshold for odor detection) than age‐matched males, while no sex‐specific differences were observed in mice in other age cohorts (Figure S1D).

To characterize changes in odor habituation and odor discrimination with aging, mice were presented with cotton swabs dipped in the same odorant (odor A) 3 times in a row before a new odorant (odor B) was presented. Mice normally spend less time sniffing a repeatedly presented odor due to habituation. When a new odor is presented, mice spend more time investigating it if they are able to distinguish it from the previously presented odor (Tzeng et al., 2021). Odorant pairs used in this assay were hexyl acetate versus octyl acetate (sweet, fruity smelling chemicals found in fruits and cheeses), lemon versus lime extracts, and two pools of mixed urine from two separate pairs of 6‐week‐old male mice (“A + B” and “C + D”). Mice in all age groups showed similar habituation to three presentations of hexyl acetate, lemon, and urine A + B (Figure S1E).

To check whether the mice were able to discriminate the presence of a new odor (octyl acetate, lime or urine C + D), we measured whether they spent more time investigating the new odor B versus the third presentation of odor A. Mice in all age groups had preserved discrimination for food odors; however, for urine odors, which are associated with social activity, only 5M mice were able to distinguish urines collected from different pairs of mice (Figure 1d–f). These results indicate that odor discrimination ability was lost during aging in mice. In addition, notable aging effects were observed in the investigative response to the first presentation of odor A (Figure 1g), with reduced sniffing time at 21 and 31M.

We also compared the performance of female and male mice at different ages for different pairs of odors. Major sex differences were observed in differentiating hexyl acetate from octyl acetate. Female mice in all age groups except 5M were able to distinguish these two odors while male mice at 5, 21 and 31M all failed to do so (Figure S2A). With respect to new odor investigation (namely odor A‐1), combining the sexes, mice started to show significantly decreased exploring time at 21M for lemon and urine and at 31M for hexyl acetate (Figure 1g). Further analysis comparing female and male separately (Figure S2B) indicated that young (5M) female mice generally showed more interest in investigating the new odors (urines and lemon) than aged‐matched male mice. A significant decrease of odor investigation behavior for lemon and urine was observed among female mice during aging, while this behavior was not significantly changed for male mice in any tested odor.

To compare these results to the time course of changes related with other behaviors, we performed additional assays related with motor functions and memory. In the open field test, mice showed a significant decline in vertical rearing activity with aging (Figure S3A); and this decline was significant in female but not in male mice (Figure S3B), similar as the observed decline in new odor exploration with age in these mouse cohorts. In the rotarod test, latency to fall off was longer in 5M old mice than in other age groups; this difference was statistically significant in Trial 5 (Figure S3C). This suggests a decline in motor coordination and balance with aging. No sex‐specific differences were observed in performance in the rotarod. DigiGait testing was used to analyze gait of mice on a motorized treadmill. The ataxia coefficient, defined as the difference between minimum and maximum stride length divided by average stride length, was significantly higher in 31M mice than in 13M mice (Figure S3D). This finding indicates greater step to step variability in aged mice. In addition, older mice had higher hind stance width, but other parameters, including paw overlap distance, hind paw angle, and hind‐midline distance (Figure S3D) were lower in older than in younger mice, indicating a significant change in gait with age. To characterize fear memory, mice were subjected to the fear context discrimination test. Unlike younger groups, 31M old mice could not discriminate between shock‐paired and unpaired environments (Figure S3E). There were no significant differences between female and male mice. In Y‐maze spontaneous alternation, a measure of spatial working memory, no significant differences were observed between age groups or between female and male mice (Figure S3F).

A summary of the behavioral changes with age is shown in Table 1. We found dysfunction in smelling to be among the earliest changes in behavior with aging in C57BL/6J mice, especially odor discrimination, sensitivity, and new odor investigation.

**TABLE 1:** The age group showing a difference relative to 5M group.

Age	13M	13M	21M			31M		Intact		
Behaviors	Odor discrimination	Motor coordination and balance	Odor sensitivity	Odor investigation interest	Gait	Odor detection	Fear context discrimination	Odor habituation	Travel distance	Spatial work related
Sex difference	Yes (13, 21 and 31M)	No	Yes (5M)	Yes (5M)	No	No	No	Yes (31M)	No	No

### 2.2 Metabolic changes during aging in brain regions associated with olfactory function

**FIGURE 2:**
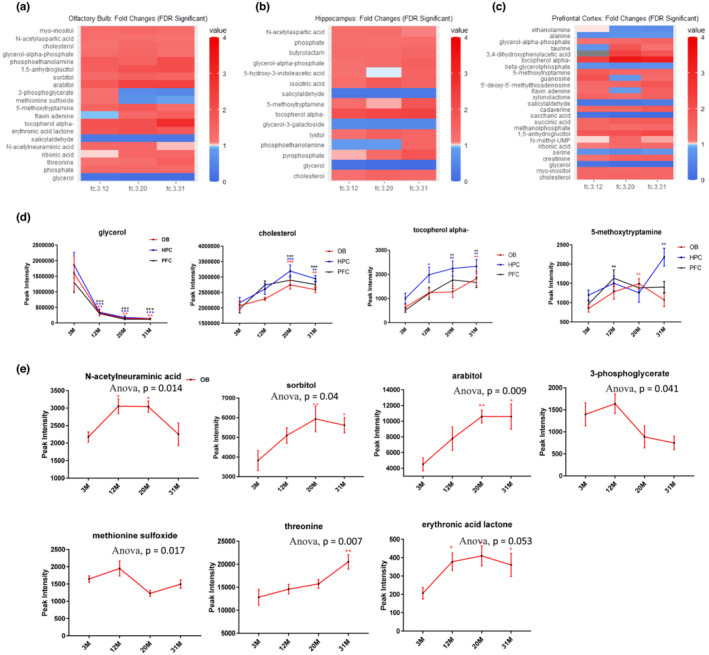
Metabolism is significantly changed in different brain regions related with olfactory function. (a–c) Heatmaps for significant known metabolites. False discovery rate (FDR) corrected *p*‐values and the fold changes as compared to 3M group. A color bar with scales for each heatmap is included. (d) Metabolites that were significantly changed in all tested brain tissue during aging. (e) Metabolites that were exclusively changed in OB during aging. One‐way ANOVA followed by corrected method of Benjamini and Yekutieliis performed as compared to 3M group in (d–e) for each type of tissue. *N* = 5 mice per group. There are 2 male and 3 female mice in 3M, 12M and 20M groups, 1 male and 4 female in 31M group. Values are mean and SEM, *p < 0.05; **p < 0.01; ***p < 0.001.

To identify metabolic changes associated with smelling dysfunction during aging, metabolites from the olfactory bulb (OB), hippocampus (HPC), and prefrontal cortex (PFC) were analyzed. In total, we identified 127 known and 226 unknown metabolites, using metabolite abundance in 3M old mice as a reference. The data in the heatmap (Figure S4A) summaries the fold‐change of all known and unknown metabolites with age. We mainly focused on the analysis of known metabolites. Specifically, there were 20, 15, and 25 significantly changed known metabolites in the OB, HPC, and PFC regions as compared to 3M, respectively (Figure 2a–c and Figure S4B). 6 metabolites were commonly changed across all tested brain regions while 3 were uniquely changed between the OB and HPC, 4 uniquely changed between the OB and PFC and 0 between HPC and PFC (Figure S4B). And 7 metabolites were uniquely identified as showing age‐dependent changes in abundance within OB tissue (Figure S4B).

Among metabolites commonly altered in all regions (Figure 2a–c), glycerol abundance sharply declined while the level of cholesterol and alpha tocopherol gradually increased with aging (Figure 2d). The effects of these metabolites on metabolism and health are well studied (Deocaris et al., 2008; Feringa & van der Kant, 2021; La Fata et al., 2014; Martin et al., 2014; Snell & Johnston, 2014) and their consistent changes with aging indicate potential use as aging markers for the brain. Routinely, studies have shown that abnormal cholesterol metabolism is associated with neurodegenerative disease (Feringa & van der Kant, 2021) and alpha tocopherol plays a neuroprotective role as an important antioxidant (La Fata et al., 2014), indicating a strong connection between these metabolites with the aging process. Additionally, 5‐methoxytryptamine changed with age in different patterns in these regions and it was specifically increased in the 12M PFC, 20M OB, and 31M HPC (Figure 2d). As this substance is an antioxidant and can protect against both tau hyperphosphorylation and amyloid‐beta pathology (Wang & Wang, 2006), its increase might be an adaptive response during aging to antagonize oxidative reactions and protein aggregates.

Seven metabolites were exclusively changed in the OB during aging, including sorbitol, arabitol, 3‐phosphoglycerate, methionine sulfoxide, erythronic acid lactone, N‐acetylneuraminic acid, and threonine (Figure 2e). Among these, N‐acetylneuraminic acid is the predominant sialic acid in human cells and it plays a role in preventing infection (Wasik et al., 2016). In our study, N‐acetylneuraminic acid was significantly and specifically elevated in the OB at 12M and 20M, indicating a potential OB‐specific response to immune‐related response during aging.

Finally, among the 20 changed metabolites in the OB, four of them (glycerol, sorbitol, myo‐inositol, and N‐acetylaspartic acid) function as osmolytes, which is important in cell volume adaptation (Figure S4B). As regulation of cell volume is particularly important in the central nervous system (Fisher et al., 2010), changes of these osmolytes during aging could potentially contribute to neuronal dysfunction. Interestingly, these osmolytes showed early changes in C57BL/6J mice at ages that corresponded to initial defects in olfaction. In addition, cholesterol (Ferris et al., 2017) and myo‐inositol (Haris et al., 2011) play roles in glial cell function; their increased abundance in the OB during aging may indicate involvement of glial cells. In summary, metabolites involved in oxidative stress regulation, infection, osmolyte homeostasis, and glial response may play roles in aging‐related olfactory dysfunction in mice.

### 2.3 Inflammatory responses are increased in brain regions related to olfactory function during aging

To characterize molecular mechanisms of smelling loss in aging, we performed unbiased gene expression microarray analysis on OB, HPC, and PFC tissue in the mouse cohorts used in this study. Cross‐tissue analysis showed that the number of significantly changed genes increased with age in all tested brain tissues and that there was greater overlap in the genes identified in the OB and PFC regions than in the OB and HPC regions (Figure S5A). Interestingly, among the commonly changed eight genes in OB, HPC, and PFC at 31M (*SLAMF9*, *CD52*, *C4A*, *IRF7*, *IFI27L2A*, *C4B*, *EGR2*, and *GM1987*), five of them (*SLAMF9*, *CD52*, *C4A*, *C4B*, and *EGR2*) were reported to be changed in mouse microglia during aging in Diseases/Drugs analysis through Enrichr (Holtman et al., 2015; Xie et al., 2021). These data are consistent with the later GFAP and IBA1 staining which suggested involvement of glial cells. Among the genes uniquely changed in OB (31M), many are related to sensory neurons (including *PCSK1*, *CALCA*, *BDNF*, *LYPD1*, *PCDHB14*, *FNDC9*, *DCLK1*, *ACER2*, *ADCYAP1*, *CYP26B1*, *IGFBPL1*, *VGF*, *HRH3* and *KLHDC9*) and olfactory bulb (including *PROKR2*, *IGFBPL1*, *AI854517* and *DCLK1*) by cell type analysis through Enrichr (Xie et al., 2021). The pathways exclusively changed in the OB include BDNF signaling (*DUSP4*, *XBP1*, *EGR3*, *HSPA5*, *FOSB*, *FOS*, *PTGS2*, *RAMP1*, and *ATF3*), AP‐1 transcription factor network (*FOSB*, *FOS*, *ATF3*, and *FOSL2*), and IL‐17 signaling (*FOSB*, *FOS*, and *PTGS2*) etc. Interestingly, more genes are differentially expressed with aging in the HPC than in the OB or PFC (Figure S5B). This indicates that HPC regions may be more sensitive and responsive to biological changes during aging (Hou et al., 2021).

**FIGURE 3:**
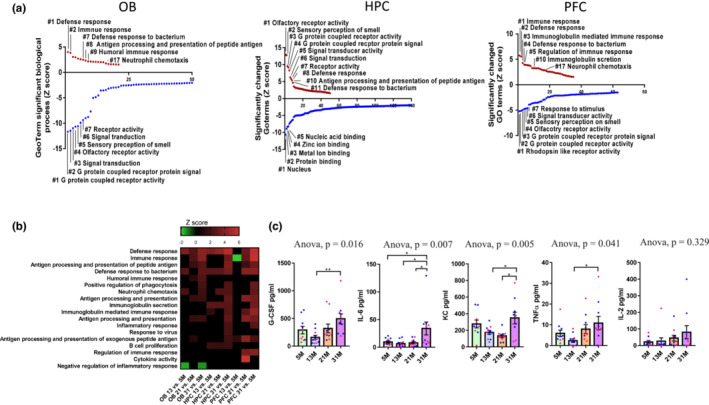
Inflammatory responses are increased during aging in brain regions related to olfactory function. (a) GO term analysis demonstrates significantly changed GO terms in 31M versus 5M mice tissues. Upregulated terms in red, downregulated in blue. (b) Microarray heatmap shows significantly changed inflammation‐related pathways in at least one comparison. *Z*‐score bar with scales for each heatmap is included, with green indicating downregulation and red, upregulation. *N* = 4 mice per group, including 2 males and 2 females. (c) Indicated cytokine or chemokine levels in mouse plasma detected by multiplex cytokine array. *N* = 10 for 12, 20 and 31‐M groups, *N* = 11 for 13 M group. One‐way ANOVA followed by Tukey's multiple comparisons test was applied for statistical analysis. Values are mean and SEM. *p < 0.05; **p < 0.01. *N* = 10–11 mice/group. Blue and red dots represent male and female mice, respectively.

Gene ontology (GO) term analysis showed that the most altered terms in the oldest (31M) versus youngest (5M) groups were related to immune response, sensory perception of smell, and G protein coupled receptor protein signal (Figure 3a). GO terms G protein coupled receptor protein signal and sensory perception of smell decreased in OB and PFC regions of older mice, while they were among the most significantly increased biological processes in the HPC. We further examined the GO term changes related to sensory functions with age in different brain tissues. As shown in Figure S5B, a significant decline in olfaction‐related terms were observed as early as 21M in OB and PFC while an increase of these terms was observed in HPC as early as 13M. Additionally, there was a time‐dependent increase in immune response‐related biological processes as the number of significantly changed terms increases with age in different regions of brains (Figure 3b), indicating activation of inflammation in OB during aging. Consistent with this, the levels of several pro‐inflammation cytokines, including G‐CSF, IL‐6, KC, and TNFα in plasma increased significantly in 31M group when compared to those at 5M (Figure 3c).

### 2.4 The olfactory bulb and hippocampus from old mice have higher DNA damage markers and lower DNA repair signals

**FIGURE 4:**
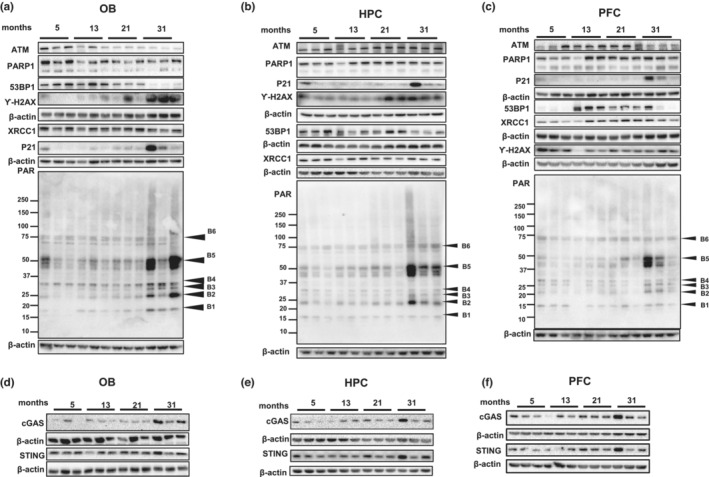
OB and HPC from old mice exhibit lower DNA damage repair signals, but higher DNA damage markers. (a–c) Immunoblots of the indicated proteins PAR, 53BP1, ATM, XRCC1, PARP1, P21, Ƴ‐H2AX and β‐actin from OB (a), HPC (b) and PFC (c). Quantification of data is shown in Figures S6B–D and S7A–C. (d–f) Immunoblots of the indicated proteins cGAS, STING and β‐actin from OB (d), HPC (e) and PFC (f). Quantification of data is shown in Figure S7D. For all blots *N* = 3 mice per group, including 1 female and 2 males.

Further analysis of the effects of aging on different cellular organelles in OB, HPC, and PFC indicates that most of these compartments were downregulated during aging, with the nucleus being the top hit across regions (Figure S6A). Because it has been postulated that declining DNA repair and increasing DNA damage contribute to aging, we examined the rate/abundance of poly ADP‐ribosylation (PARylation), a pivotal post‐translational protein modification, and the expression of DNA repair proteins in the age‐stratified mouse cohorts. As shown in Figure 4a–c and Figure S6B–D, PARylation increased significantly in OB and HPC of 31M mice. Further quantification of PARylation levels of major individual proteins at different molecular sizes showed that although OB, HPC, and PFC shared certain commonly changed PARylation sites (B3), the PARylation levels of unidentified proteins B1 and B4 were exclusively changed in OB during aging (as shown in Figure 4a–c and Figure S6B–D). Further studies are needed to unveil the identity and function of these proteins.

Interestingly, despite the significant increase of PARylation in the OB, the protein levels of ATM, 53BP1 and XRCC1, which play important roles in DNA repair, were down regulated in OB tissue from the 31M group compared to the younger groups (Figure 4a and Figure S7A). Insufficient expression of these proteins would potentially cause defects in DNA repair and accumulation of DNA damage, which is consistent with the higher expression levels of p21 and γ‐H2AX (Figure 4a). In the HPC (Figure 4b and Figure S7B) a significant decrease in 53BP1 and increase in γ‐H2AX was observed. In the PFC, lower expression of 53BP1 and higher expression of γ‐H2AX was detected in 5M old mice, with no significant decrease in expression of other DNA repair proteins. Expression of p21 in PFC was higher in the oldest mice than in younger groups (Figure 4c and Figure S7C).

We examined protein levels of cGAS and STING, which have been shown to induce senescence and inflammation after activation by DNA damage, processes that we found evidence for in aged mice. cGAS protein was significantly increased in OB tissue from 31M mice; nonsignificant trends were seen in HPC and PFC. STING protein was not significantly altered during aging (Figure 4d–f and Figure S7D).

Together our results suggest increased protein PARylation and altered expression of DNA repair proteins in the OB of the oldest cohort of mice. We postulate that these changes might lead to increased DNA damage and inflammation, and eventually lead to olfactory dysfunction.

### 2.5 Activation of microglia and astrocytes in the aging olfactory bulb

**FIGURE 5:**
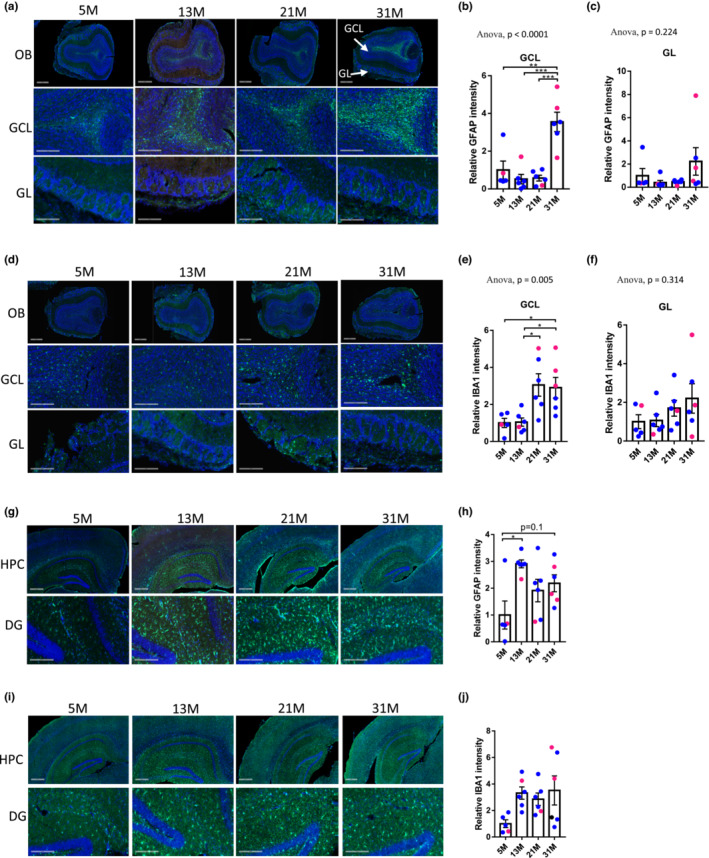
Expression of GFAP and IBA1 are increased with aging in OB tissue. (a) Representative images of GFAP (green) and DAPI (blue) staining in OB from different age groups. (scale bars: 500 μm in images from the first row, 200 μm in the second and third rows). (b, c) Quantification of GFAP signal in GCL (b) and GL (c) layers of OB. (d) Representative images of IBA1 (green) and DAPI (blue) staining in OB from different age groups (scale bars: 500 μm in images from the first row, 200 μm in the second and third rows). (e, f) Quantification of relative IBA1 signal in GCL (e) and GL (f) layers of OB. (g) Representative images of GFAP (green) and DAPI (blue) staining in HPC and DG (dentate gyrus) from different age groups (scale bars: 500 μm in images from the first row, 200 μm in the second row). (h) Quantification of relative GFAP signal in HPC from different age groups. (i) Representative images of IBA1 (green) and DAPI (blue) staining in HPC and DG from different age groups (scale bars: 500 μm in images from the first row, 200 μm in the second row). (j) Quantification of relative IBA1 signal in HPC from different age groups. For all graphs, *N* = 5–6 mice per group; blue and red dots represent male and female mice, respectively; one‐way ANOVA followed by Tukey's multiple comparisons test was applied for statistical analysis. Values are mean and SEM. *p < 0.05; **p < 0.01; ***p<0.001.

Since the microarray and WB analyses revealed increased inflammation in OB and HPC of old mice, we further investigated whether there was activation of microglia and astrocytes, two important types of glial cells in the brain. Astrocytes comprise the most abundant population of glia in the mammalian brain. They not only provide structural support for the brain but also play a variety of essential functions in synapse development, neurotransmitter homeostasis, and neurogenesis etc. An active inflammatory state of astrocytes is associated with increased expression of GFAP and is routinely observed in brain diseases and neuropathology (Siracusa et al., 2019). Here, we found that GFAP was highly expressed in the granule cell layer (GCL) and glomerular layer (GL) of OBs. Elevated GFAP expression was seen in 31M mice selectively in the GCL layer (Figure 5a–c). Since the GFAP signal was mainly detected in the center area of GCL (Figure S8A), we further analyzed the change of GFAP with age by examining GCL in the center and outer areas to determine whether GFAP staining was homogeneously increased throughout the GCL. The results in Figure 5b and Figure S8A,B showed that there was the significant accumulation of GFAP particularly in the center region of GCL at 31M, indicating that the increase of GFAP with aging in GCL is not homogeneous. The expression of IBA1, a marker for microglia, was significantly higher in the GCL layer of the 21M and 31M groups than in the 5M and 13M groups. There were no significant differences of IBA1 in the GL of OB among the different age groups (Figure 5d–f). Higher expression of GFAP was also detected in the HPC regions from older groups than 5M group (Figure 5g,h). Changes in IBA1 within HPC tissue did not reach statistical significance (Figure 5i,j).

### 2.6 NR treatment increases lifespan and partially restores olfactory function in aged mice

**FIGURE 6:**
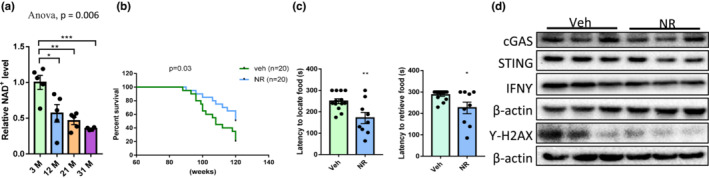
NR treatment increases lifespan and olfactory functions of old wild type mice. (a) Relative NAD^+^ level in OB tissue. *N* = 4–5 mice per group. One‐way ANOVA followed by Tukey's multiple comparisons test was applied for statistical analysis. (b) Lifespan of mice with/without NR supplementation. *N* = 20 mice per group, all males. *p*‐values compared with vehicle calculated using log‐rank test. (c) Time to start digging and grasp the food and in the buried food test. Unpaired *t* test was applied. *N* = 8–13 mice per group, all males. (d) Immunoblots of the indicated proteins cGAS, STING and β‐actin from OB. *N* = 3 mice per group, all males. Quantification of data is shown in Figure S9D. Values are mean and SEM. *p < 0.05; **p < 0.01; ***p<0.001.

NAD^+^ is an important metabolite that is associated with aging and aging‐related neurodegeneration. The decline in NAD^+^ may result in inadequate DNA repair, mitochondrial dysfunction, and inflammation. Supplementation of NAD^+^ through NR or nicotinamide mononucleotide improves the health span and neuronal function in animal models of Alzheimer's disease (Hou et al., 2018), ataxia telangiectasia (Yang et al., 2021), and xeroderma pigmentosum (Fang et al., 2014). To identify how NAD^+^ level changes specifically in the OB during aging, levels of NAD^+^ and total NAD (NADt, defined as the sum of NADH and NAD^+^) were measured and compared between samples from different ages. With increasing age there was a significant decline in NAD^+^ (Figure 6a) and a similar nonsignificant trend in NADt (Figure S9A).

To investigate whether supplementation of NAD^+^ with NR preserves smelling function in aging, 24M old mice were provided with water containing 12 mM NR for 8 months before smelling‐related behavior tests were performed. Mice dosed with NR had a longer lifespan than the water‐only control group (Figure 6b). In the buried food test, mice treated with NR spent significantly shorter time before digging at the buried food site and finding the hidden food, indicating better odor detection (Figure 6c). However, NR did not improve performance in tests of odor discrimination or sensitivity (Figure S9B,C). These smelling behavior tests together indicate that NR supplementation can partially improve olfactory function in aged mice. Treatment with NAD^+^ also altered expression of γ‐H2AX, cGAS, STING and IFNγ in older mice. Expression of these three proteins was lower in NR‐treated mice than in control mice (Figure 6d and Figure S9D). This is consistent earlier study (Hou et al., 2021) showing that treatment with NAD^+^ reduces neuroinflammation and stimulates DNA repair.

## 3 DISCUSSION

Here, we systematically performed a multi‐domain behavioral test battery to characterize olfactory, cognitive, and locomotor changes in wild type healthy C57BL6/J mice of four ages. We demonstrate that smelling loss potentially is an early biomarker of aging compared to memory and motor behaviors. In addition, we show that inflammation and DNA damage confer risks to olfaction decline by using metabolomics, transcriptomics, and signal protein profiling approaches. Finally, we found that supplementation of NAD^+^ through NR preserves olfaction and improved longevity in aged mice.

Human olfactory function typically begins to decline in the sixth decade of life (Mullol et al., 2012; Zhang & Wang, 2017) and increase in prevalence with age (Schubert et al., 2011). Olfactory dysfunction refers to a decrease in the ability of odor detection, odor discrimination, and odor memory (Tzeng et al., 2021). The olfactory system is phylogenetically conserved in humans and mice, sharing remarkable similarities in odorant receptor proteins, organization of the peripheral and central olfactory pathways, and odor‐related behaviors (Ache & Young, 2005). Furthermore, the same odorants have similar attraction to mice and humans, illustrating a component of odor preference conserved across the two species (Mandairon et al., 2009). Therefore, mice represent a valuable model for understanding molecular mechanisms underlying both aging‐ and neurodegeneration‐related changes in the olfactory system. Here, we used mice at different ages ranging from mature adult, middle age, old age to extreme old age. This setup is different from previous studies, which mainly have only compared young adult (2–6M) and aged animals (22–28M) (Enwere et al., 2004; Fahlstrom et al., 2012; Forster et al., 1996) or between young adult and middle aged animals (Shoji et al., 2016). Here, olfactory function was evaluated across multiple domains using a battery of behavioral tests: odor detection by the buried food test, odor sensitivity by measuring the threshold of odorant concentration required to elicit investigation, odor habituation and discrimination by using three different pairs of similar odors in the habituation–dishabituation test. Among these olfaction domains, the earliest difference was observed in discriminating urine, as only 5M mice were able to discriminate pooled urine samples collected from different groups of mice. For other odor pairs, all age groups retained intact ability. This result indicates that loss of odor discrimination during aging is odorant selective, and that the use of social odors may generate more sensitive results in future mouse studies. Odor sensitivity testing indicated that 5M and 13M mice shared the same threshold, while mice at 21M and 31M demonstrated higher ones. Despite the lower odor sensitivity for 21M mice, they had similar performance as 5M mice in the buried food test, suggesting that the former test may be better at detecting subtle impairments. Significant impairments in the buried food task were only observed in 31M mice. Odor habituation remained intact in all age groups. Additionally, a significant decrease in investigation time was observed in older mice (21M and 31M) in three tested odors when compared to the young adult, suggesting a decline in olfaction‐induced exploration. Altogether, our data showed that odor discrimination and sensitivity were among the earliest changed domains in aged mice. The design of more sensitive odor discrimination tests by choosing proper odor pairs may be useful in detecting early and subtle smelling changes in human aging and neurodegenerative diseases.

Besides the effects of age on olfactory function, we also evaluated sex differences in all the olfactory tests among mice at different ages. Table 1 indicates that odor discrimination, motor learning and balance, odor sensitivity and investigation may come earlier than memory loss in aging mice. Generally, female mice demonstrated better performance than male mice in odor sensitivity and discrimination, which is consistent with the observation in humans that women performed better than men in all age groups and male olfaction declined significantly faster with age than women (Sorokowski et al., 2019). However, women and men reach a comparable level of decline in the oldest (80–97 years old) age group (Schubert et al., 2011). These data suggest that C57BL/6J mice can serve as a model to explore sex differences found in the human olfactory function.

We investigated whether smelling loss comes earlier than other behavioral changes by comparing the timeline of the changes. Motor function and cognition, which are two important functions significantly affected by aging, are monitored by a battery of behavioral tests. Among these, the earliest change was observed in the rotarod test as 13M and older mice displayed a significantly shorter latency to fall off when compared to 5M mice on the last of five trials, indicating a decline in motor coordination and balance. Some of the gait features started to show differences at 21M, like hind paw angle and hind stance width, notably few prior studies have linked these parameters with aging. Among the gait features which start to change at 31M, the ataxia coefficient is relevant to human aging as an index of step‐to‐step variability that predicts risk of falling (Callisaya et al., 2010). No significant differences were observed between age groups or between female and male mice in the memory tests, the latter of which is consistent with research showing no sex differences in human memory (McDougall et al., 2014).

Pathologic conditions mostly disorganize metabolic processes, resulting in changes that can be observed as metabolic signatures (Botas et al., 2015). Metabolic profiles of OB tissue from older and younger mice significantly showed differences. Though the biological functions of many metabolites remain unknown, several metabolites which were highly related with brain biology were significantly changed in a systematic or region‐specific way during aging. For example, glycerol significantly decreased with aging among all the three brain regions. Glycerol is recommended as a dietary supplement during caloric restriction and a practical intervention for “healthy aging” (Deocaris et al., 2008; Snell & Johnston, 2014). It has a number of beneficial effects, including lifespan extension, stress resistance, and mitochondrial activity enhancement (Deocaris et al., 2008; Snell & Johnston, 2014). Thus, the attenuated glycerol concentration might be a potential marker of aging. Additionally, increased cholesterol levels in all tested brain regions during aging indicated an abnormal cholesterol level, which is associated with neurodegenerative disease (Feringa & van der Kant, 2021). Alpha tocopherol plays a neuroprotective role as an important antioxidant (La Fata et al., 2014), and its elevated level in aging may be an indicator of increased oxidative stress. Myo‐inositol is considered a possible marker for amyloid‐related pathology (Voevodskaya et al., 2019). Increased myo‐inositol has been linked to AD, gliomatosis cerebri, recovering hypoxia, and other human diseases (Haris et al., 2011). The increased myo‐inositol in aged OB tissue in our case potentially indicates early pathological changes. Additionally, both cholesterol and myo‐inositol are related to glial cells (Feringa & van der Kant, 2021; Haris et al., 2011; Petrov et al., 2016). In our study, we observed significant increases of myo‐inositol and cholesterol as early as 12M and 21M in OB, respectively, which suggests early changes in glial cells and potentially indicating smelling changes.

Inflammation and glial activation induce loss of smelling and atrophy of the OB (Hasegawa‐Ishii et al., 2019, 2020). Astrocytes and microglia, two important types of glial cells in brain, were evaluated by GFAP and IBA1 expression, respectively. Our results revealed elevated levels of both GFAP and IBA1 in the granular cell layers of OB from older groups (21M and 31M). Microglial activation in aged OB tissue indicated active inflammatory responses in brain and is taken as a hallmark of brain pathology (Dheen et al., 2007). Consistently, unbiased gene expression analyses showed that the immune‐related responses were among the most up‐regulated GO terms while G protein coupled receptor signaling were the most down‐regulated GO terms in 31M OB samples. Altogether, activated glia and upregulated immune responses contribute to OB dysfunction during aging.

The nucleus and nucleosomes were the most significantly affected cellular compartments in OB tissues from 31M versus 5M. Accordingly, aged OB tissue had reduced DNA repair capacity and higher protein levels of Ƴ‐H2AX and p21. In our study, we found that the NAD^+^ level significantly declined with age in OB tissue (Figure 6a and Figure S9A). As an essential substrate for PARPs to produce PAR chains in DNA repair, diminished NAD^+^ levels are associated with Xeroderma Pigmentosum complementation (Fang et al., 2014) and Ataxia Telangiectasia (Yang et al., 2021). Supplementation of NAD^+^ through NR reduced expression of markers for DNA damage and inflammatory signaling in OB, and improved smelling behavioral test performance in aged mice. Additionally, it significantly increased the lifespan of wild‐type C57BL/6J mice, indicating that NAD^+^ supplementation not only provides benefits to smelling but also for general health.

Collectively, our findings show that the earliest smelling decline in mice can be observed at 13M via odor discrimination for urine samples, while impairment in odor sensitivity and investigation are evident by 21M. Consistently, we observed molecular and protein‐level changes as early as 13M. Specifically, the levels of glycerol, tocopherol alpha, 5‐methoxytryptamine, N‐acetylneuraminnic acid, and NAD^+^ began to decline as early as 13M in brain regions related with smelling; proteins important for DNA repair, including ATM and PARP1 decreased from 13M; significant changes in olfaction related GO terms were found in HPC as early as 13M, while changes in OB and PFC regions occurred later at 21M. These results also indicate that HPC may be a brain region related to olfaction changes in early aging. This idea is consistent with our GFAP staining, showing that the earliest ages showing increases were 13M and 31M for HPC and OB, respectively. Significant aging‐related molecular changes were detected in OB, including increases in DNA damage markers, inflammation related pathways, IBA1 and GFAP staining, cGAS protein level, and plasma cytokine levels.

In the present study, we have performed a systematic characterization of smelling loss in non‐ pathological aging. This includes a multi‐age timeline of changes within different odor domains, sex differences, and a comparison with other age‐related behavioral and molecular changes. While it is not our main goal to distinguish between olfactory dysfunction linked to non‐pathological aging and disease‐associated pathology in the present study, our results are helpful in addressing this question by showing the baseline of smelling loss in normal aging and serves as reference when a disease mouse model, on a similar background, are evaluated. Additionally, our findings indicate that both DNA damage repair and inflammation contribute to olfaction decline, which is partially preserved by NAD^+^ supplementation. Thus, our data support the concept that olfactory dysfunction has the potential to be an early biomarker of aging, and NR treatment can preserve olfaction in aged individuals.

## AUTHOR CONTRIBUTIONS

X.D., B.Y., D.L.C., and V.A.B. designed experiments. B.Y., Q.C., S.G. and R.M. performed animal treatment and behavior tests. B.Y. and D.F. collected the mice samples. X.D. and X.C. performed Western blot and histology. X.D, D.L.C. and Chris Morrell performed metabolism analysis. X.D. performed NAD^+^ measurement. Y.Z. and D.L.C. performed microarray analysis. X.D. and S.G. wrote and B.Y., D.L.C., M.P.M., and V.A.B. revised the manuscript. All authors contributed to writing the final manuscript.

## CONFLICT OF INTEREST STATEMENT

V.A.B. had CRADA arrangements with ChromaDex but receives no personal benefit. All others declare no competing interests.

## Supporting information


**Appendix S1:** Supporting Information

**Appendix S2:** Supporting Information

## Data Availability

The data that supports the findings of this study are available in the supplementary material. The accession number for the raw and processed microarray data reported in this paper is GSE204966.
