# Supplementary material for: Loss of smelling is an early marker of aging and is associated with inflammation and DNA damage in C57BL/6J mice
Source: Aging Cell. 2023 Feb 27;22(4):e13793. doi: 10.1111/acel.13793 (PMC10086518; doi:10.1111/acel.13793)
Supplement: Supplementary file 1 — Appendix S1: Supporting Information [file ACEL-22-e13793-s002.docx]

**SUPPORTING INFORMATION**

**Supplemental Experimental Procedures**

**Animals.** C57BL/6J mice came from either Jackson Laboratories or the NIA Aged Rodent Colonies and were habituated to housing at the National Institute of Aging (NIA) Intramural Research Program for at least 1 month prior to experimentation. All mice were maintained on a standard NIH diet (T.2018SX.15 Global 18% Protein Extruded Rodent Diet, sterilizable) with food and water available ad libitum. Animals were maintained on a 12-h light/dark cycle, with all testing performed during the light cycle. The animals were group housed if possible. All animal experiments were performed using protocols approved by the appropriate institutional animal care and use committee of the NIA, 361-OSD-2023.

**Antibodies.** Primary antibodies used included anti-PARP1(#9542S, Cell Signaling); p-p53 (CS9284S, Cell Signaling), cGAS (#31659, Cell Signaling); STING (#13647, Cell Signaling), XRCC1 (ab1838, Abcam), ATM (ab201022, Abcam); p53 (60283-1-Ig, Proteintech IFRγ (15365-1-AP, Proteintech), β-actin (sc-1616, Santa Cruz); p21(610234, BD Biosciences), PAR (4336-BPC-100, Trevigen), IBA1 (ab178846, Abcam), and GFAP (Z033401-2, DAKO).

**Buried Food Test.** Tests were performed based on methods described [[12](#_ENREF_12)]. All mice were habituated in home cages to 45mg reward pellets (5TUL formulation; TestDiet, St Louis, MO) used for the assessment 2 days prior to testing. Mice were fasted for 18 hr prior to testing and then habituated for 60 minutes in a clean 14 × 35 cm cage filled with 3 cm of woodchip bedding. After habituation, the mouse was removed from the test cage and placed out of sight while a reward pellet was buried under the bedding, just above the bottom of the cage. The mouse was then placed back into the testing cage opposite the buried pellet and given 10 minutes to find the buried food. The length of time the mouse took to start digging in the correct location and to grasp the pellet in its forepaws were recorded by a scorer blinded to conditions.

**Olfactory Discrimination Test.** Tests were adapted from published protocols [[12](#_ENREF_12), [13](#_ENREF_13)]. Each mouse was placed in a 19 × 27 cm clean cage containing a thin layer of corncob bedding to absorb urine. Mice were presented with a sequence of odorants soaked into a 1.5cm long cotton tip attached to a 15cm wooden handle, which was suspended from a hole in the center of the cage lid. The cotton tip was 5.5 cm above the cage floor, a height that required extension of the head but not rearing on hind limbs for close investigation.

Food extracts (McCormick & Company; Hunt Valley, MD), chemicals (Sigma Aldrich; St Louis, MO), or freshly collected mouse urine were diluted in deionized (DI) water or mineral oil at a concentration (1:1000) that was dilute enough to require close approach for investigation but strong enough that all ages of mice could readily detect. Prior to testing, each mouse underwent a 30-minute habituation with a vehicle-scented cotton tip. After this, the mouse was presented three times serially with vehicle and then three times with odor A. On a final trial, the mouse was presented with odor B. The inter-trial interval was one minute and each trial lasted two minutes. A scorer blinded to conditions recorded sniffing behavior, defined as orientation of the mouse’s head toward the cotton tip and nose within 2 cm. Biting and climbing of the cotton tip or wooden handle were not scored as sniffing. Mice that did not show any sniffing behaviors for the cotton tip three times in a row were excluded from analysis.

**Olfactory Sensitivity/Threshold Test.** Procedures were similar to odor discrimination, with the following changes. On each day of testing, concentrated orange extract was first diluted in DI water (1:100) and was serially diluted 10-fold. After habituation, the mouse being tested was presented with cotton applicators dipped in DI water for four trials. The mouse was then presented with serial trials of cotton applicators dipped in odor dilutions, ordered from the weakest to the strongest. The lowest concentration of odorant detected was determined when the sniffing time of diluted orange odor was significantly higher than that of the water. Mice that did not show any sniffing behaviors were excluded from analysis.

**Rotarod.** Rotarod software (MED-Associates; St Albans, VT) started rotation at 4 RPM and accelerated over the 5-min testing period to 40 RPM. Animals from the same cage (up to 4) were placed on the rotarod and monitored during the trial. If a mouse fell off during the trial, the rotarod apparatus and software recorded this time and the mouse was quickly returned to its home cage. Mice were tested 3 times with an intertrial interval of 60 minutes.

**Open Field.** Open field testing was conducted in a 44 x 44 x 30 cm clear Plexiglas arena, which was enclosed in a dimly lit sound attenuating cubicle. All animals were placed into this device and monitored for 10 minutes with use of the Activity Monitor software (MED-Associates).

**DigiGait.** Mice were habituated to the DigiGait (Mouse Specifics Inc, Framingham, MA) apparatus for 5 minutes. Animals were recorded by collecting digital images at 80 frames per second with a highspeed video camera located beneath a transparent treadmill moving at a speed of 15 cm/s. Gait parameters were analyzed R software. Mice that did not walk on the belt were excluded from analysis.

**Fear Context Discrimination.** Automated Video Freeze software (Med Associates) was used to measure freezing behavior. On day one, mice were habituated to the chamber for 2 min, followed by three electric foot shocks (2s; 0.5 mA) separated with 1-min intervals. On day two, mice were first tested for 5 min in the same testing chamber presented identically to conditioning on day one. Approximately 3 h later, mice were placed for 5 min into a modified chamber with plastic floor and ceiling inserts, darkened (near infrared only) lighting, and different odors.

**Western Blots.** Olfactory bulb (OB), hippocampus (HPC), and prefrontal cortex (PFC) were collected and lysed in radioimmunoprecipitation assay (RIPA) buffer (Cell Signaling Technology, MA, USA) containing protease inhibitors (Bimake) and phosphatase inhibitors (Bimake). Samples were sonicated at power 4 for 10 s on ice by an ultrasonic cell disruptor followed by centrifugation at 14 000 g for 30 min and collection of supernatants. Proteins were separated on 4–12% Bis-Tris gel (Thermo Fisher Scientific) and transferred onto a PVDF membrane. After blocking in 5% milk (Bio-Rad) in TBST (20 mM Tris–HCl pH7.4, 0.14 mM NaCl, 0.1% Tween^®^ 20) for 1 h at room temperature, membranes were incubated with a primary antibody at 4°C overnight. After incubation with the HRP-conjugated secondary antibody for 1 h at room temperature, the signals were detected using a PharosFX plus Molecular Imager (Bio-Rad). Band intensity was determined using FIJI-ImageJ software.

### Immunofluorescence. Mice were anesthetized with isoflurane and then were perfused with cold PBS buffer following with 4% paraformaldehyde (PFA) in PBS. After perfusion, spray the head with 70 % ethanol. Tent the scalp with large forceps and remove with a single transverse cut made with large scissors. Stabilize the anterior skull with large forceps placed between eyes and nose. Insert small dissection scissors into foramen magnum and carefully cut the skull as far anteriorly as possible. Use the small forceps to carefully pry each half of the skull away from the brain. Then gently lift the brain out of the skull and place the brains in 4% PFA for 24h before equilibration in 30% sucrose for another 48h at 4°C. A series of 30-μm coronal sections in OCT were collected in a 6-well plate for further experiments. Brain slices were incubated in blocking buffer (5% BSA in PBS) for 30 min at RT, permeabilized in 0.2% Triton X-100 for 5 min at RT, incubated overnight with primary antibody at 4 °C, and incubated in secondary antibodies for 1 h at RT. Nuclei were counterstained with DAPI. Slides were scanned using Leika Aperio Versa 200 and analyzed using ImageScope and FIJI-ImageJ software.

**Microarray.** RNA from OB, HPC and PFC were purified and inspected as reported previously [[14](#_ENREF_14)]. Briefly, RNA was purified with PureLink™ RNA isolation kit following manufacturer's protocol (Thermo) and its quality were evaluated using a 2100 Bioanalyzer (Agilent Technologies). Microarray analysis was performed by the Gene expression and Genomics core facility (NIA) and analyzed using DIANE 6.0 software. Two female and two male mice were used for each group. Pathways/GO terms were considered significant if they had more than three genes and a t-test p-value <0.05 with an FDR of not >0.3.  The detailed data analysis methods were described previously [[15](#_ENREF_15)]. The raw data is available through [GSE204966](https://www.ncbi.nlm.nih.gov/geo/query/acc.cgi?acc=GSE204966).

**Cytokine detection.** Mouse eye bleeds were collected in EDTA-treated tubes. Plasma was obtained by collecting the supernatant of blood after centrifugation. Samples were then flash frozen until detection. Plasma cytokines and chemokines were detected by using 31-Plex Cytokine/Chemokine array (Eve Technologies).

**Metabolite measurement.** Perfused, flash-frozen brain tissue was later thawed for primary metabolism by GC-TOF MS, performed by Metabolomics Service Core, UC Davis. Analysis included sugars, TCA metabolites, amino acids, hydroxyl acids such as lactate, free fatty acids, aromatics, polyamines, nucleosides, monophospho nucleotides. Primary metabolite analysis was done with an Agilent 6890 GC equipped with a Gerstel dual MPS injector, using a Leco Pegasus IV time of flight mass spectrometer with Leco ChromaTOF software. Raw files were processed upon data acquisition and run through in-house database, Binbase. The data are provided in relative peak heights for more precision of low abundant metabolites. Results were normalized using mTIC (the sum of the peak heights of the known metabolites).

For each metabolite there were five measurements at each of the four ages (3, 12, 20, 31 months). We determined if there were a significant difference in the mean at later ages as compared to 3 months. For each metabolite a one-way ANOVA was performed. The p-value for the test of equality of all means was saved along with the p-values comparing the later months to 3 months. To guard against multiple testing, these three p-values were also corrected using the false discover rate (FDR) method. In addition, the fold change in the mean as compared to 3 months was computed for each age groups, 12, 20, and 31 months. R was used for data analysis and using both the uncorrected p-values, the FDR corrected p-values, and the fold changes, heatmaps are constructed for all metabolites as well as for significant and FDR significant metabolites. For the significant known metabolites, a cluster analysis was conducted to determine which metabolites group together. The resulting heatmap is ordered by these clusters. Finally, graphs were constructed for known metabolites that have either a significant ANOVA p-value or significant comparisons relative to 3 months. The raw data is available in Supplementary Excel 1.

**NAD^+^ measurement.** Perfused, flash-frozen OB from male mice were later thawed and tested with a commercially available NAD^+^/NADH assay kit (no. ab65348; Abcam) according to the manufacturer’s protocol.

**Statistics.** GraphPad Prism 6.0 was used for the statistical analysis. The data are shown as the mean ± SEM with P < 0.05 considered statistically significant. Group differences were analyzed with one-way or two-way ANOVA for comparison among multiple groups. The two-tailed paired t test was applied for comparisons between two variables for the same mouse.

**Supplementary figures**

**
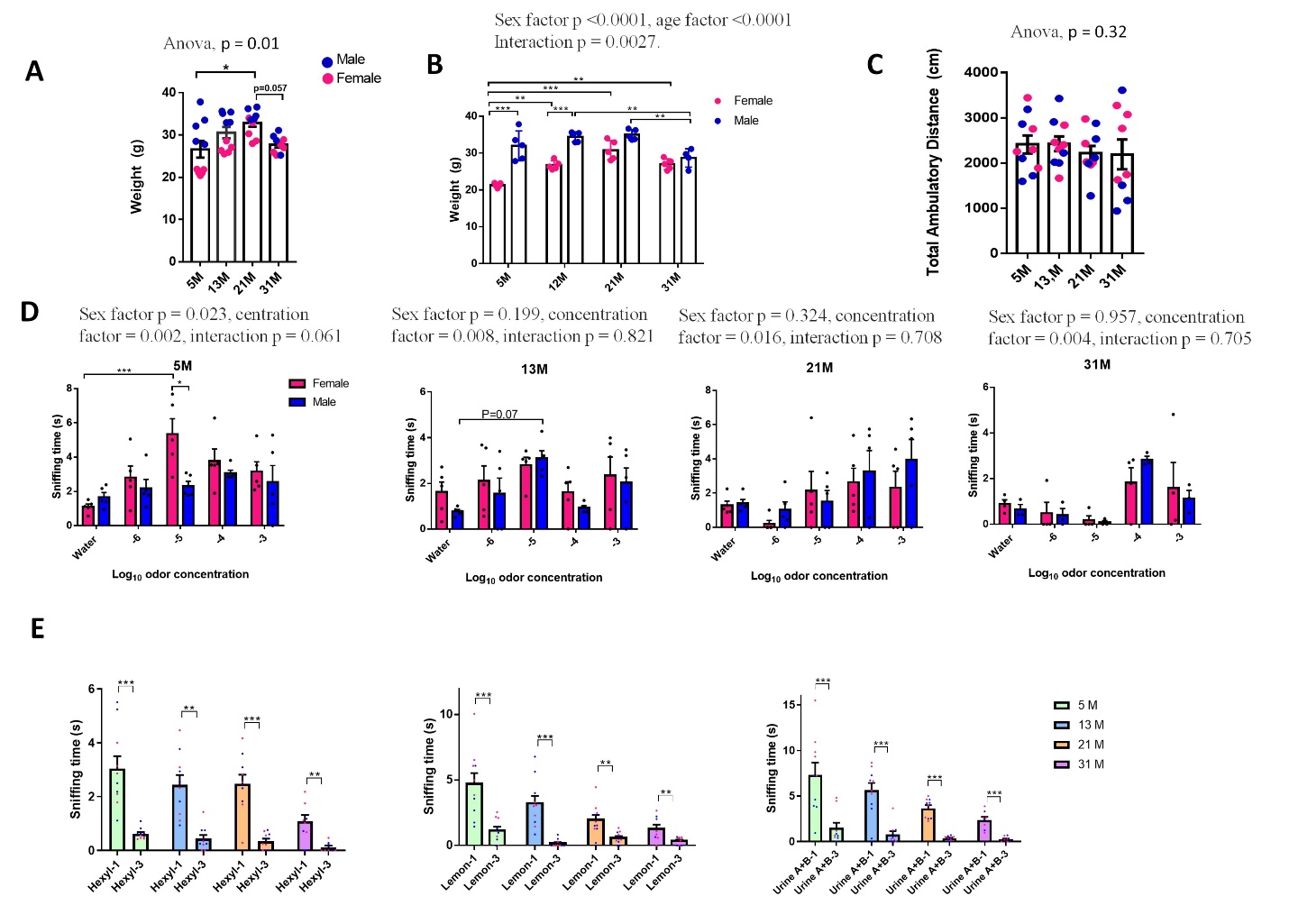
**

**Figure S1 Smelling-related behavioral changes during aging.** A. Body weights of mice. One-way ANOVA followed by Tukey's multiple comparisons test was applied for statistical analysis. N = 9-10 mice per group. B. Body weights of male and female mice at different age groups. Two-way ANOVA followed by Tukey's multiple comparisons test was applied for statistical analysis. N = 3-5 mice per group. C. Total distance traveled in the open field test. One-way ANOVA followed by Tukey's multiple comparisons test was applied for statistical analysis. N = 9-10 mice per group. D. Odor sensitivity test of male and female mice at different age groups. Two-way ANOVA followed by Tukey's multiple comparisons test was applied for statistical analysis. N = 3-5 mice per group. E. Odor habitation test. Paired t-tests (two-tailed) were performed to compare the sniffing time between the first and third presentation of odor A (A-1 and A-3). N = 8-10 mice per group. Values are mean and SEM.*P < 0.05, **P < 0.01, ***P < 0.001. N = 3-5 mice per group. Male and female mice are represented by blue and red dots, respectively, in all graphs.


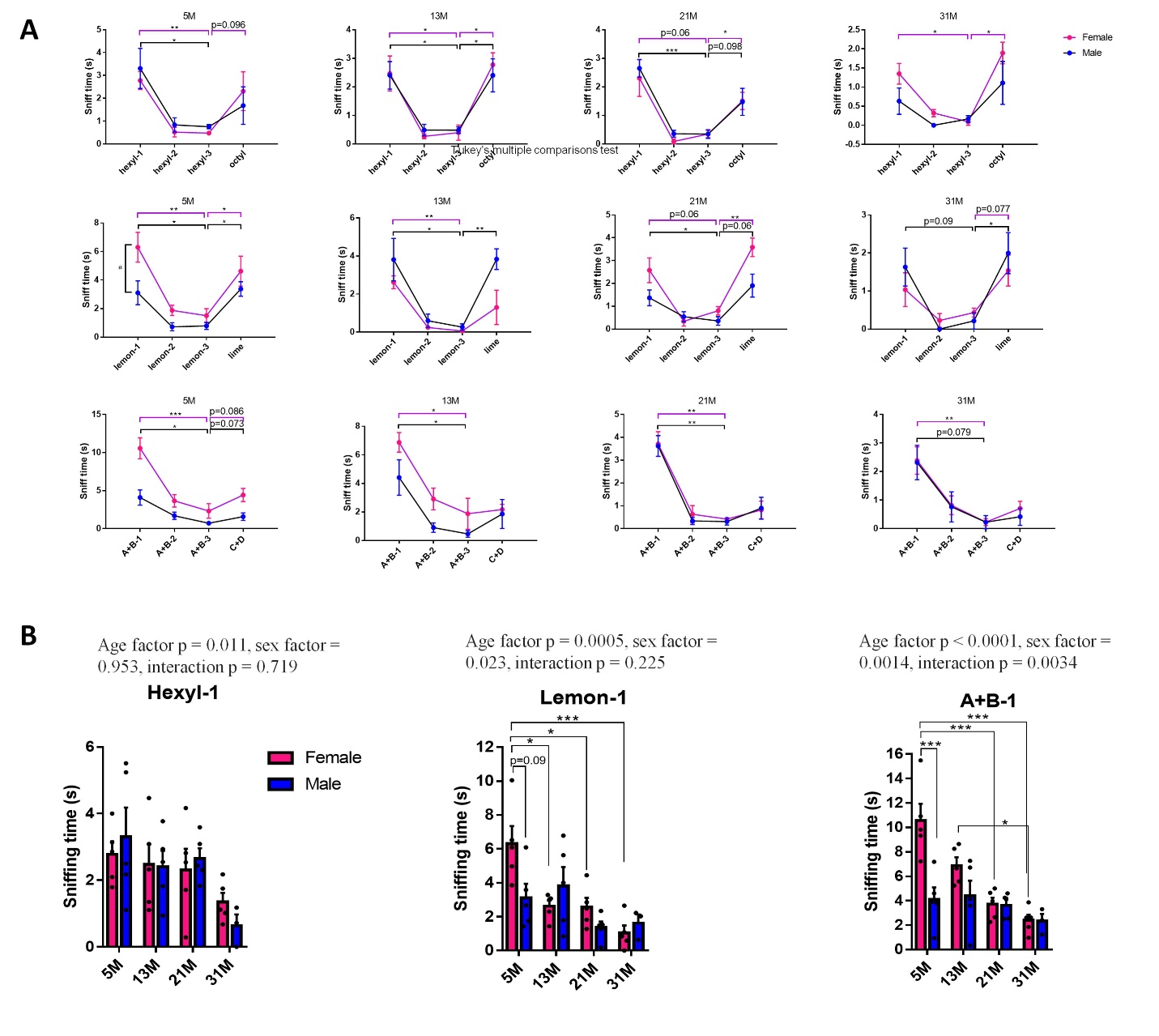


**Figure S2 Change of odor habituation and new odor investigation during aging.** A. Odor habituation -dishabituation testing of male and female mice at different age groups. Paired t-test (two-tailed) was applied to compare odor A-1 to odor A-3, and odor A-3 to odor B for male and female, respectively. N = 3-5 mice per group. B. New odor investigation of male and female at different ages. Two-way ANOVA followed by Sidak's multiple comparisons test was applied for statistical analysis. Values are mean and SEM.*P < 0.05, **P < 0.01, ***P < 0.001. N = 3-5 mice per group. The graphs and statistical analysis for male and female mice are represented in blue and red, respectively, in all graphs as indicated.


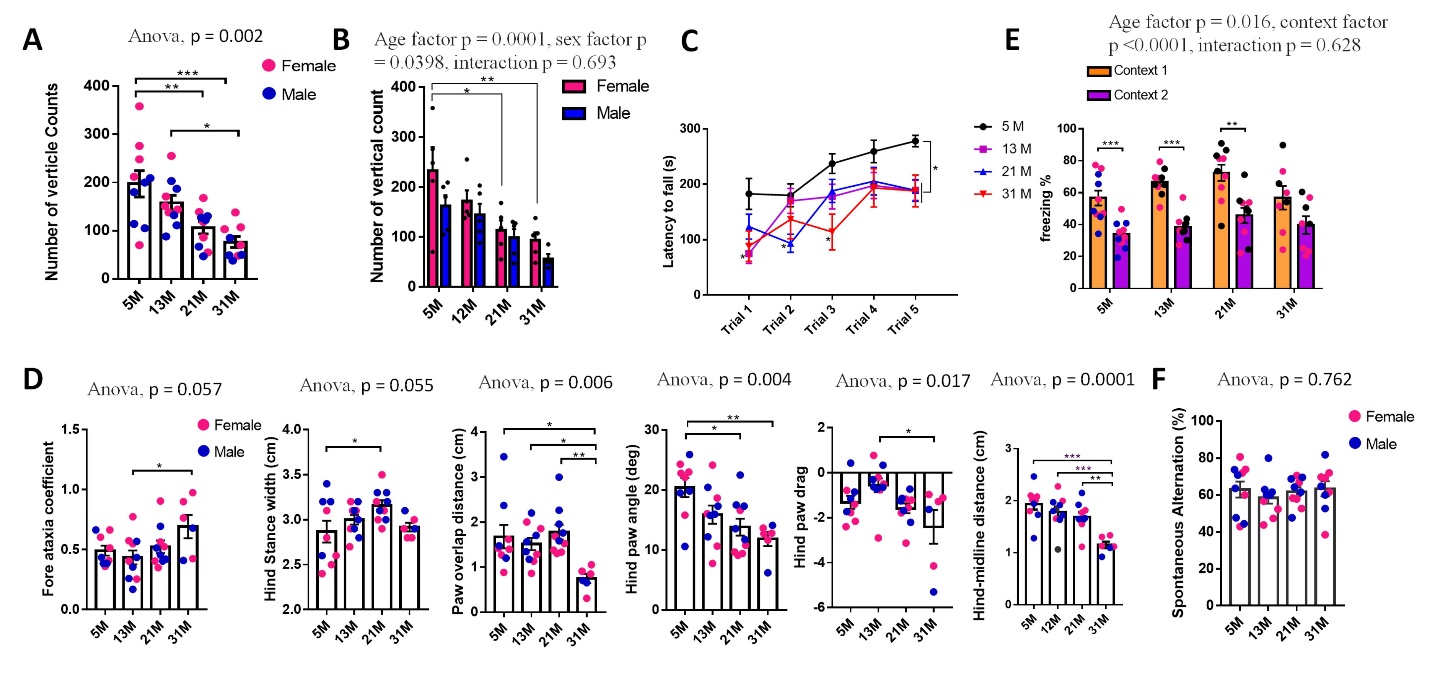


**Figure S3 Non-smelling-related behavioral changes during aging.** A-B. The number of vertical counts in open field test for mice at different ages without (A) and with the consideration of sex factor (B). One-way ANOVA followed by Tukey's multiple comparisons test and two-way ANOVA followed by Tukey's multiple comparisons test were applied for A and B, respectively. N = 9 -10 mice per group. C. Latency to fall in the rotarod test. One-way ANOVA followed by Tukey's multiple comparisons test was applied to compare the latency to fall among different age groups at the trial 5. N = 8 -10 mice per group. D. Different gait parameters in DigiGait test. N = 6-10 mice per group. One-way ANOVA followed by Tukey's multiple comparisons test was applied for statistical analysis. E. Percent freezing in fear conditioning test. Two-way ANOVA followed by Sidak's multiple comparisons test (comparison within each age group) was applied for statistical analysis. N = 8 -10 mice per group. F. Rate of spontaneous alternation in the Y-maze test. One-way ANOVA followed by Tukey's multiple comparisons test was applied. Values are mean and SEM.*P < 0.05, **P < 0.01, ***P < 0.001. N = 9 -10 mice per group. Male and female mice are represented in blue and red, respectively, in all graphs.


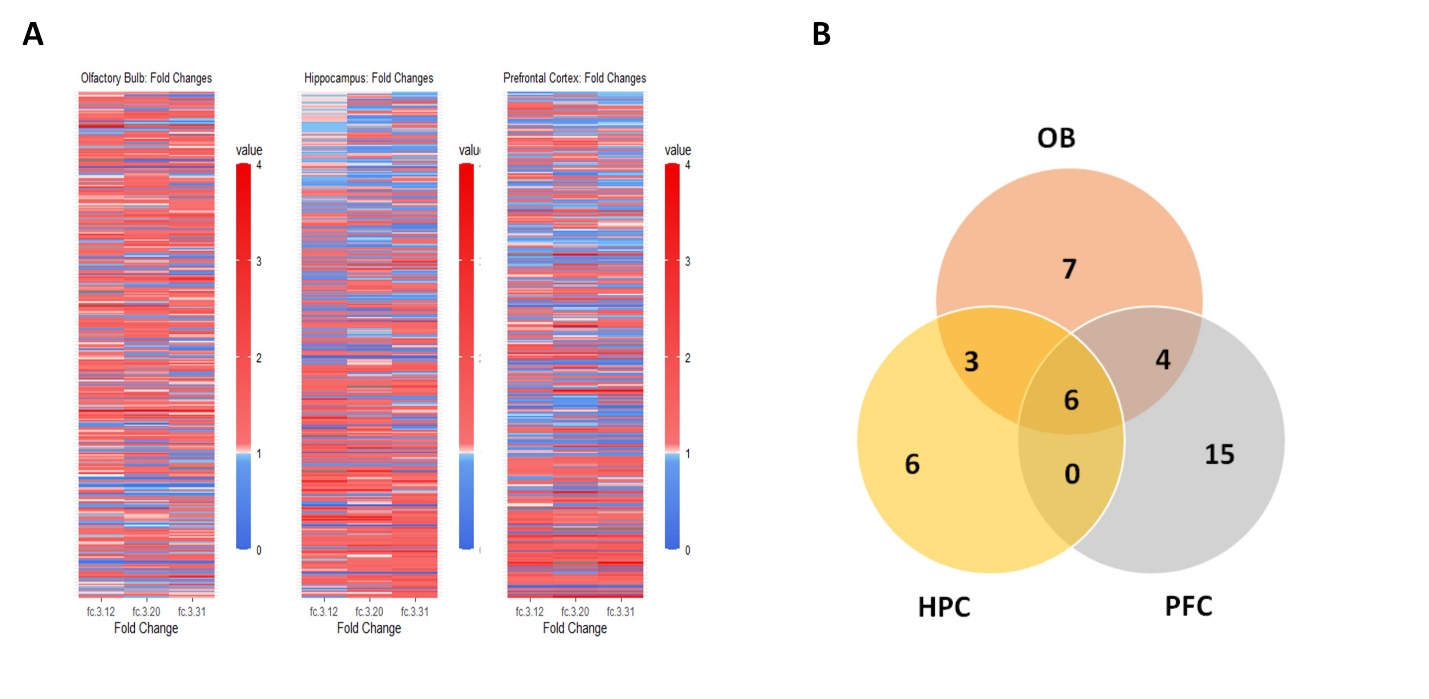


**Figure S4 Metabolites changes in aging.** A. Using fold changes, heatmaps are constructed for all analyzed metabolites. A color bar with scales for each heatmap is included. B. Venn diagram showing the number of significantly changed known metabolites based on false discover rate (FDR) corrected p-values on the fold changes as compared to 3M group. N = 5 mice per group. There are 2 male and 3 female mice in 3M, 12M and 20 M groups, 1 male and 4 female in 31M group.


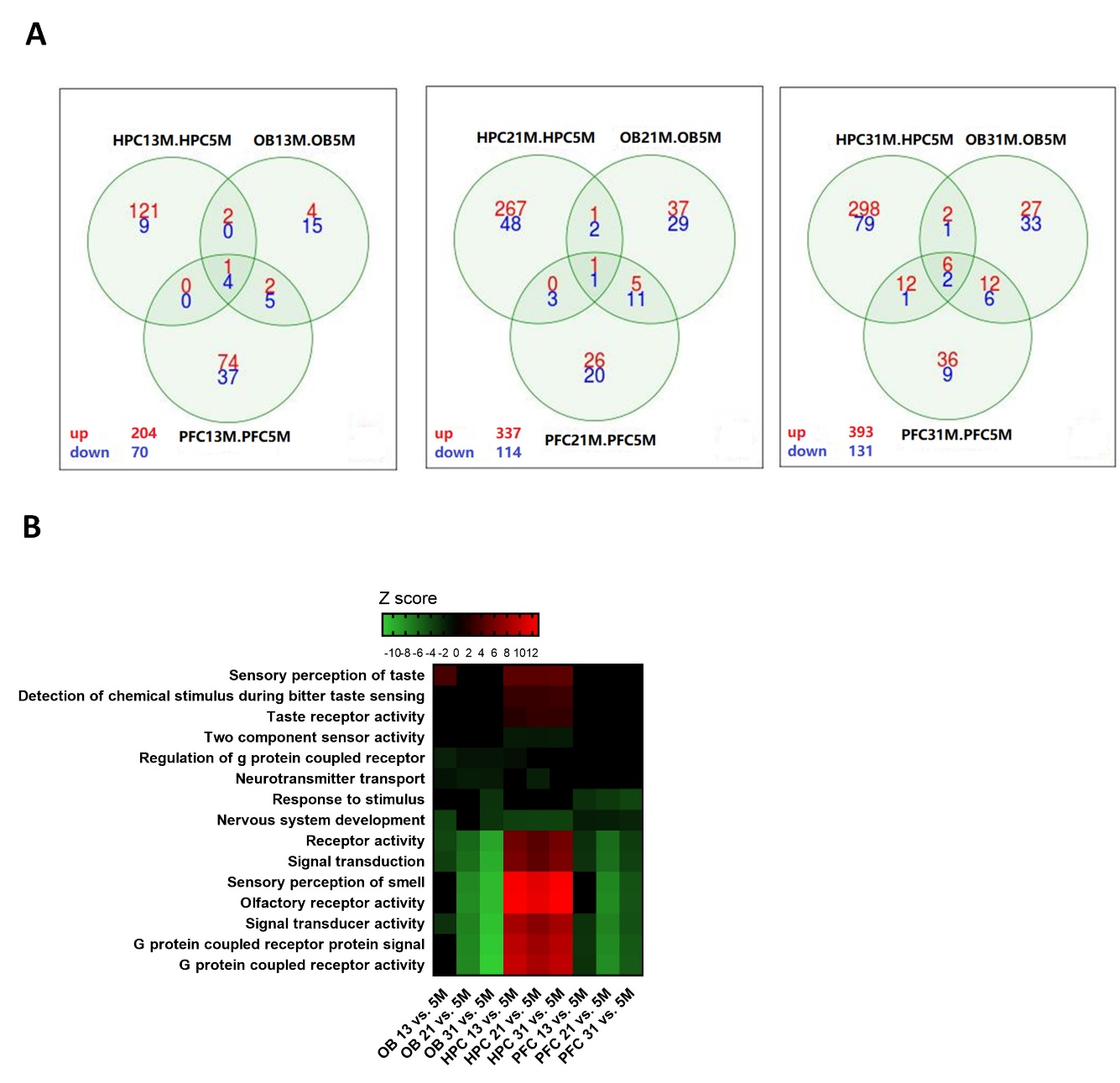


**Figure S5 Microarray analysis of genes and GO term changes in brain tissues during aging.** A. Venn diagram showing the number of significantly changed genes in different brain region as compared to respective tissue at 5M. B. Heatmap of olfaction-related GO terms that were significantly changed in at least one group of comparison. Z-score bar with scales for each heatmap is included. N = 4 mice per group, including 2 males and 2 females.

**Figure S6 Evaluation of DNA damage in brain tissues during aging.** A. Heatmap of different organelle-related GO terms. N = 4 mice per group, including 2 males and 2 females. B-D. Quantification of PARylation level of all proteins and individual proteins shown in Figure 4A-C. One-way ANOVA followed by Tukey's multiple comparisons test was applied for statistical analysis. N = 3 mice per group, including 1 female and 2 males. Values are mean and SEM.*P < 0.05, **P < 0.01, ***P < 0.001.


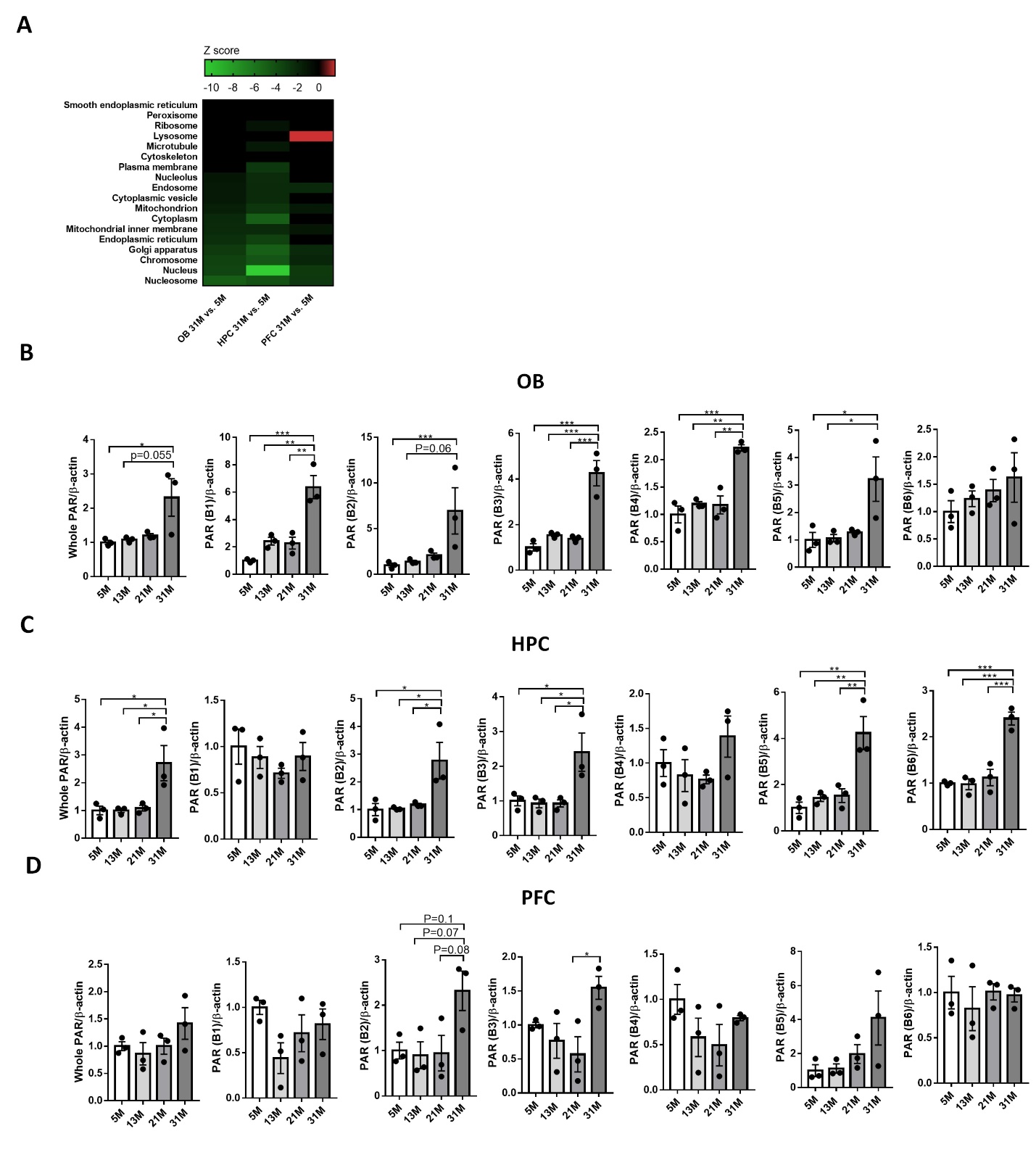


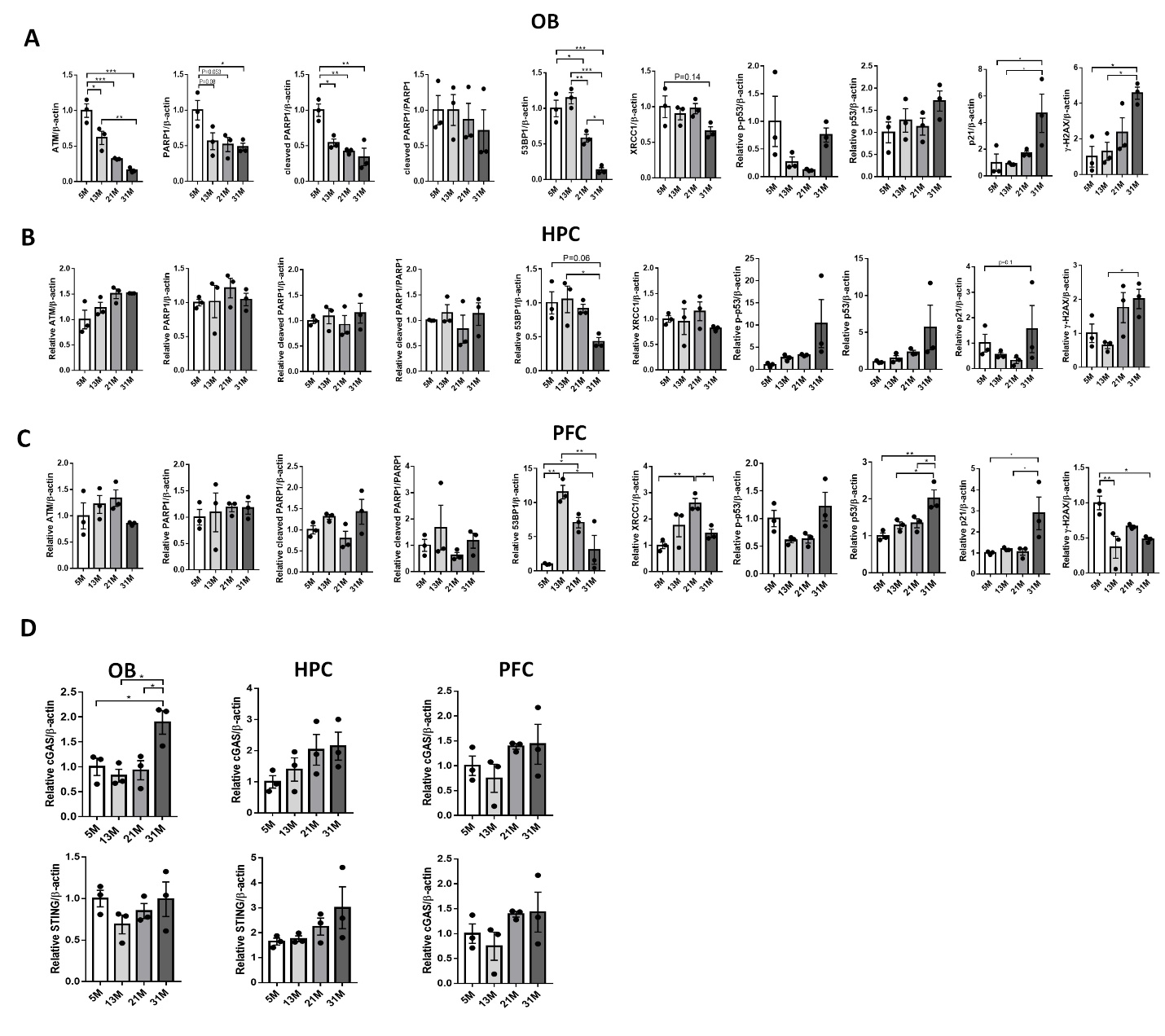


**Figure S7 Quantification of proteins related to DNA repair, damage marker and inflammation.** A-C. Quantification of DNA repair proteins and DNA damage markers in Figure 4A-C. D. Quantification of cGAS and STING level in Figure 4D-F. One-way ANOVA followed by Tukey's multiple comparisons test was applied for statistical analysis. N = 3 mice per group, including 1 female and 2 males. Values are mean and SEM.*P < 0.05, **P < 0.01, ***P < 0.001.


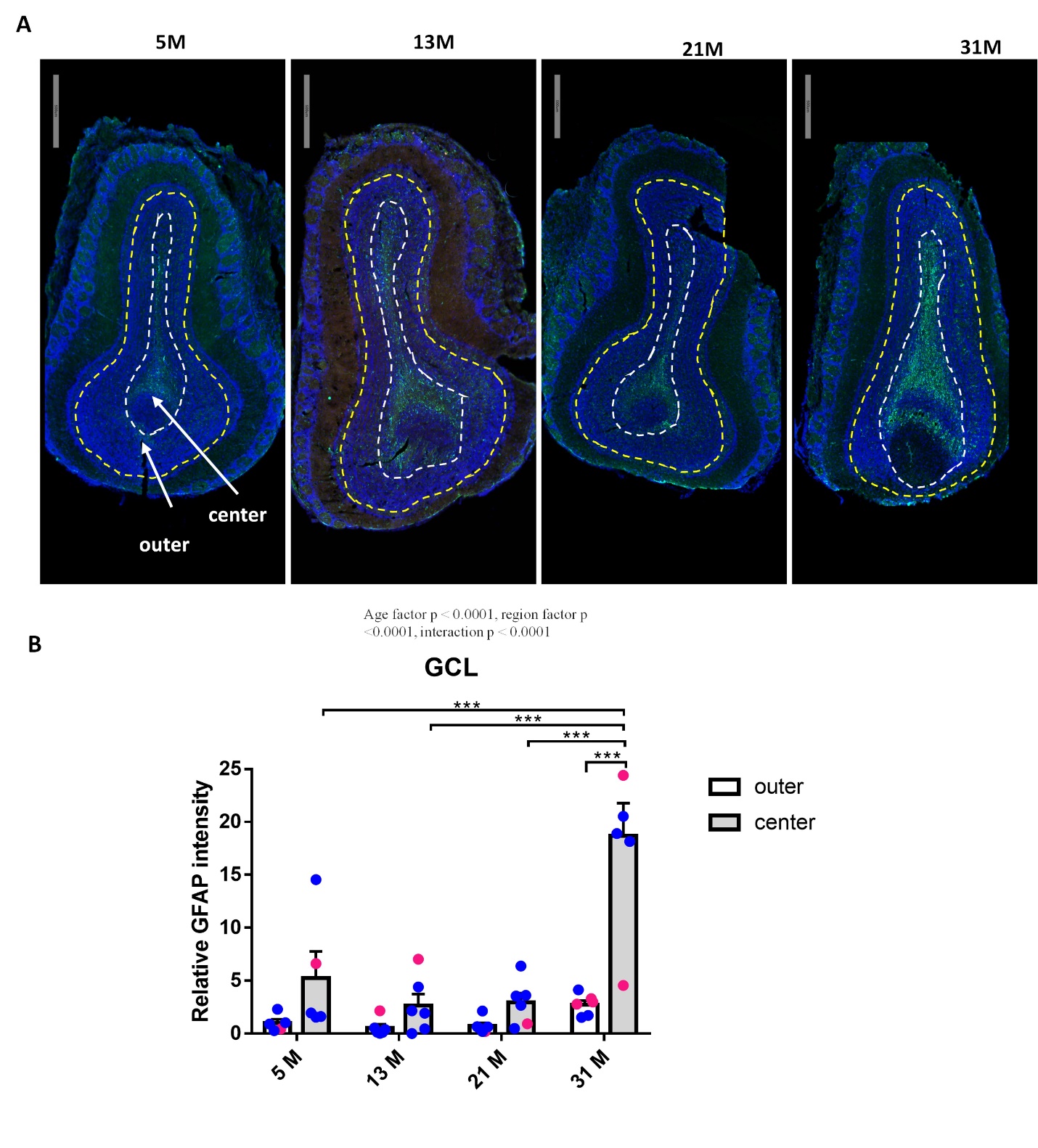


**Figure S8 GFAP staining in the center and outer region of GCL. A.** Representative images showing the center and outer regions of GCL in OBs from different age groups. Scale bars: 500 µm in images. B. Relative GFAP intensity as compared to that of outer region in OB from 5M groups. N = 5-6 mice per group; blue and red dots respectively represent male and female mice; Two-way ANOVA followed by Tukey's multiple comparisons test was applied. Values are mean and SEM. *P < 0.05, **P < 0.01, ***P < 0.001.


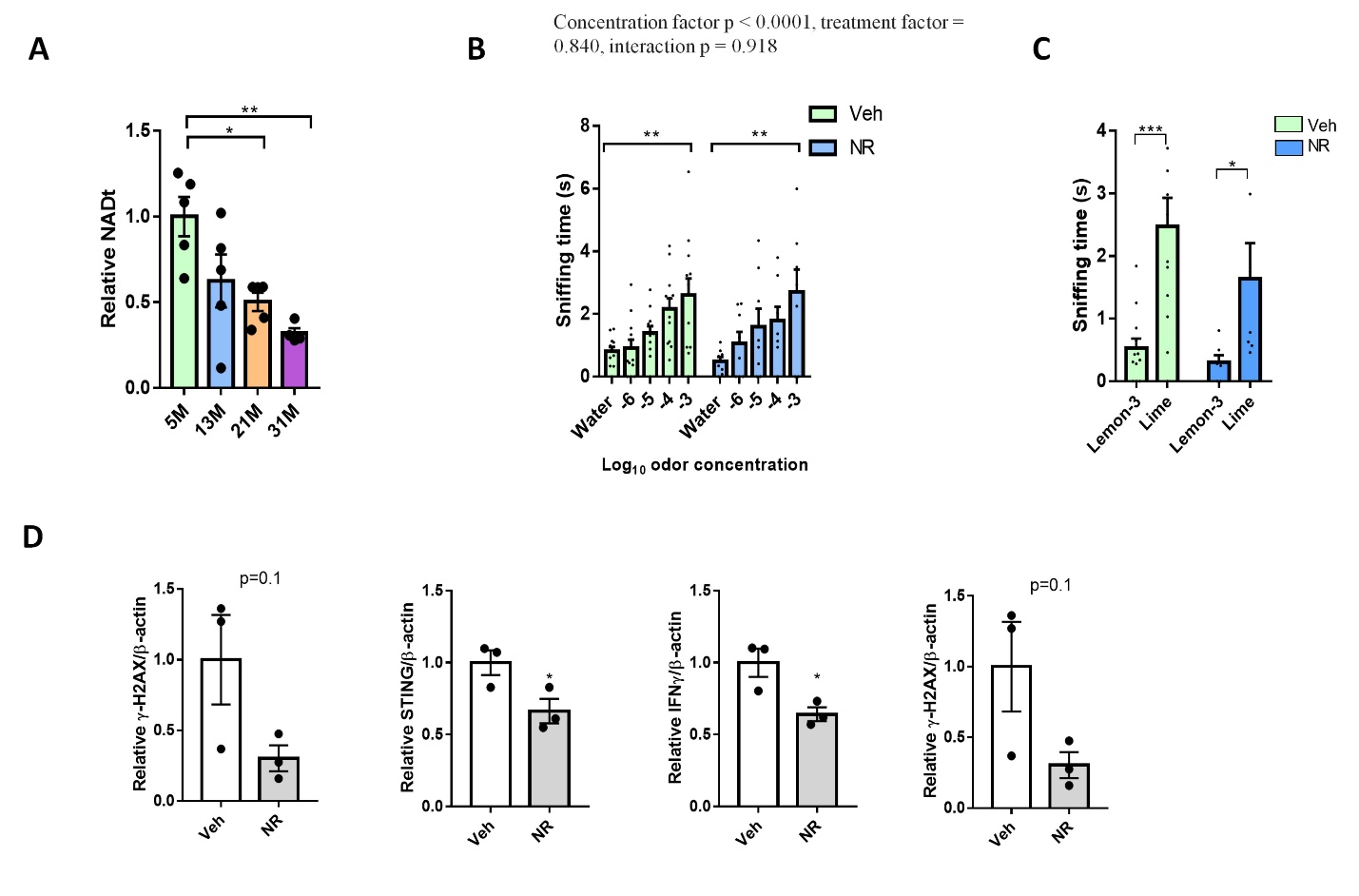


**Figure S9 Effects of NAD+ supplementation on smelling behavior and inflammation. A.** Relative tNAD level in OB tissue. N = 4-5 mice per group. One-way ANOVA followed by Tukey's multiple comparisons test was applied for statistical analysis. B. Graphic representation of odor sensitivity test. Two-way ANOVA followed by Tukey's multiple comparisons test (comparison within each treatment group) was applied for statistical analysis. N = 8-12 male mice per group. C. To characterize odor habituation and discrimination, paired t-tests were performed comparing the sniffing time between odors A-1 and A-3, and between odors A-3 B for mice with/without NR treatment. N = 7-13 male mice per group. D. Quantification of relative cGAS, STING and IFN-γ of OB tissue from mice with/without NR supply. Unpaired t-test was applied for statistical analysis. Values are mean and SEM.*P < 0.05, **P < 0.01, ***P < 0.001. N = 3 male mice per group.
